# Peroperative Findings of the Middle Turbinate in 50 Patients With Chronic Sinusitis Who Underwent Total Spheno-Ethmoidectomy

**DOI:** 10.1155/DTE.5.1

**Published:** 1998

**Authors:** T. D. Morre, P. A. R. Clement, G. Noussios

**Affiliations:** 1 Department of Otorhinolaryngology Head and Neck Surgery University Hospital Free University Brussels, Laarbeeklaan 101 Brussels 1090 Belgium; 2 Heerbaan 123 Londerzeel B-1840 Belgium

## Abstract

This study describes the peroperative endoscopic findings about the size, shape and mucosal changes of the middle turbinate in patients with chronic sinusitis who underwent total spheno-ethmoidectomy. Results confirmed the middle turbinate to be a useful landmark in performing extensive sinus surgery. The most frequent change due to chronic inflammation seems to be polypous degeneration followed by hyperplastic mucosa. Anatomical variations, being paradoxically bent turbinate and concha bullosa, are not seen frequently.


*Diagnostic and Therapeutic Endoscopy*, Vol. 5, pp. 1–8
Reprints available directly from the publisher
Photocopying permitted by license only

© 1998 OPA (Overseas Publishers Association) N.V.
Published by license under
the Harwood Academic Publishers imprint,
part of The Gordon and Breach Publishing Group.
Printed in Singapore.

### Peroperative Findings of the Middle Turbinate in 50 Patients with Chronic Sinusitis Who Underwent Total Spheno-Ethmoidectomy

T.D. MORRE*, P.A.R. CLEMENT and G. NOUSSIOS

*Department of Otorhinolaryngology, Head and Neck Surgery, University Hospital, Free University Brussels, Laarbeeklaan 101, 1090 Brussels, Belgium*

*(Received 10 April 1998; In final form 21 April 1998)*

**This study describes the peroperative endoscopic findings about the size, shape and mucosal changes of the middle turbinate in patients with chronic sinusitis who underwent total spheno-ethmoidectomy. Results confirmed the middle turbinate to be a useful landmark in performing extensive sinus surgery. The most frequent change due to chronic inflammation seems to be polypous degeneration followed by hyperplastic mucosa. Anatomical variations, being paradoxically bent turbinate and concha bullosa, are not seen frequently.**

*Keywords:* Middle turbinate, Spheno-ethmoidectomy, Sinus endoscopy

### INTRODUCTION

The middle turbinate or concha media is the medial appendage of the lateral nasal wall and lays over the bulla ethmoidalis and in most cases over the hiatus semilunaris and the uncinate process [1]. This turbinate is for most authors an important landmark in endoscopic sinus surgery [1,2]. Others on the other hand present the middle turbinate as a poor landmark when sometimes prior to surgery or polypoid disease, the middle turbinate can vary from its usual position [3]. The authors observed the middle turbinate during surgery in 50 chronic sinusitis patients. The aim of the present study is to study the mucosal changes and the anatomic variations of the middle turbinate in patients with extensive inflammatory sinus disease needing total spheno-ethmoidectomy.

### MATERIALS AND METHODS

Fifty patients (32 men and 18 women, mean age 48 years) with symptomatic chronic rhinosinusitis

* Corresponding author. Heerbaan 123, B-1840 Londerzeel, Belgium. Tel.: +32 52 30 64 00. Fax: +32 2 477 64 23.

with no response to medication were scheduled for total spheno-ethmoidectomy under general anesthesia from 1993 to 1996. All operations were recorded on videotapes. Ten out of the 50 patients had massive nasal polyposis. To evaluate the middle turbinate 4 mm rigid 30° nasal panoview endoscopes were used. A $\frac{3}{4}$in U-matic format with high image quality has been used for all recordings [4]. The tapes were viewed by the same person. Of the 50 patients each side of the nose, i.e. 100 sides were examined and photographed from the videotapes. From all the 100 middle turbinates mucosal aspect and anatomic variants were noted. According to age, the population was divided into three groups: less than 40 years, between 40 and 50 years and the third group older than 50 years of age. The mucosal aspect was divided into normoplastic, hyperplastic and polypous. Anatomical variations considered were concha bullosa and paradoxical curvature of the middle turbinate.

### RESULTS

All patients underwent total spheno-ethmoidectomy. Out of this 64% of the patients were between 40 and 60 years of age (Table I). The different types of mucosal aspects encountered and the anatomic variations of the middle turbinates are presented in Table II. Predominantly

TABLE I Patients variables

| Variable | Number of patients (/50) |
|---|---|
| Age | |
| < 40 years | 11 |
| 40–60 years | 32 |
| >60 years | 7 |
| Sex | |
| Female | 18 |
| Male | 32 |

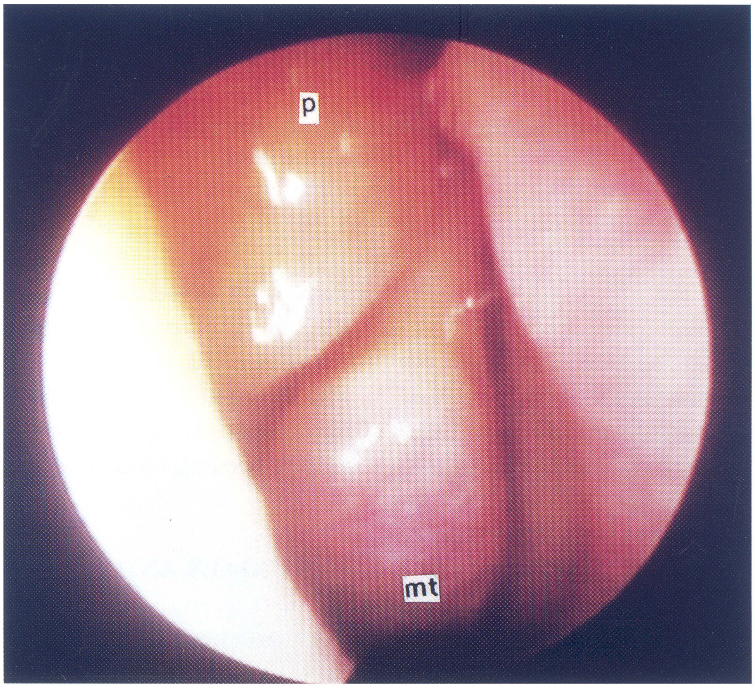

FIGURE 1 Polypous middle turbinate.

altered mucosal aspect of the middle turbinate i.e. polypous (43%) (Fig. 1) (mt = middle turbinate, p = polyp, ap = antrochoanal polyp) and hyperplastic (26%) (Fig. 2) was encountered whereas 31% of normoplastic turbinates (Fig. 3) were distinguished. Bilateral polypous mucosa was seen in 17 patients whereas 6 patients had bilateral hyperplastic mucosa and 10 patients proved to have bilateral normoplastic mucosa. As for the anatomic variations, surprisingly more paradoxical curvatures (13%) (Fig. 4) of the middle turbinate were seen than conchae bullosa (4%) (Fig. 5). Only three sagittally clefted middle turbinates were observed unilaterally (Fig. 6). All conchae bullosa appeared to be unilateral and 3 patients had bilateral paradoxical curvature of the middle turbinate. In 3 patients a unilateral antrochoanal polyp was encountered always on the left side (Fig. 7). Massive nasal polyposis was seen bilaterally in 8 patients and unilaterally in 2 patients (Fig. 8). In spite of massive nasal polyposis, the middle turbinate still remained normoplastic in 4 patients which confirms the middle turbinate as an important landmark in spheno-ethmoidectomies (Fig. 9).

TABLE II Results

| Variable | Number of examined middle turbinates (/100) |
|---|---|
| Mucosa | |
| Normoplastic | 31 |
| Hyperplastic | 26 |
| Polypous | 43 |
| Anatomic variations | |
| Concha bullosa | 4 |
| Paradoxical curvature | 13 |
| Sagittally clefted | 3 |

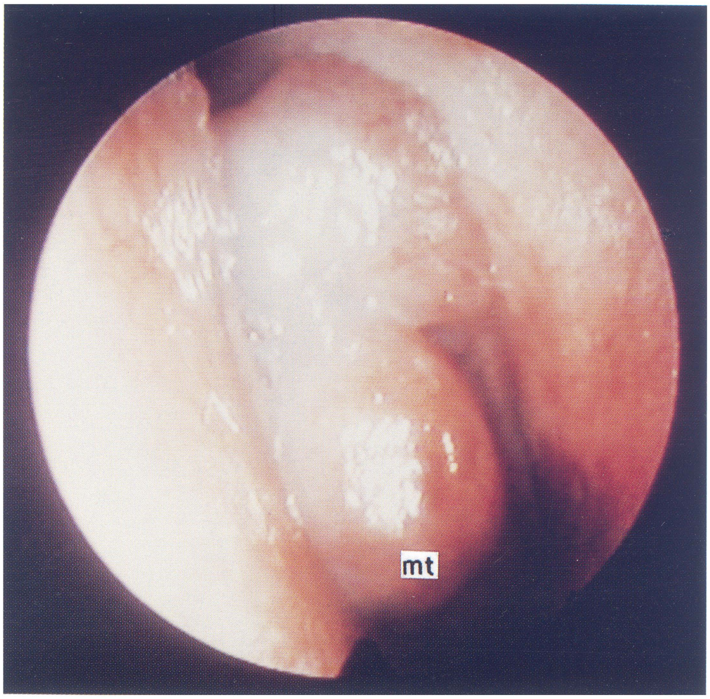

FIGURE 2 Hyperplastic middle turbinate.

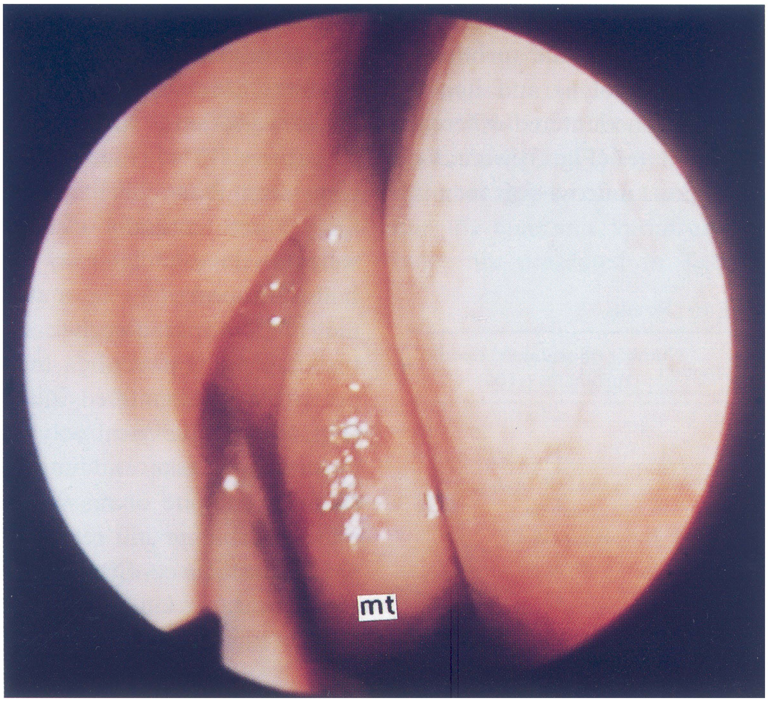

FIGURE 3 Normoplastic middle turbinate.

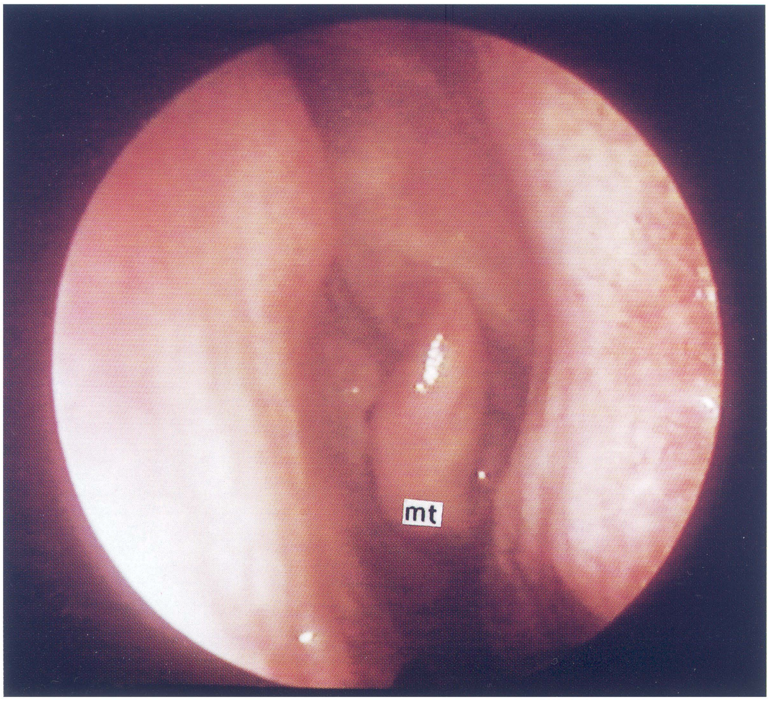

FIGURE 4 Paradoxical curvature of the middle turbinate.

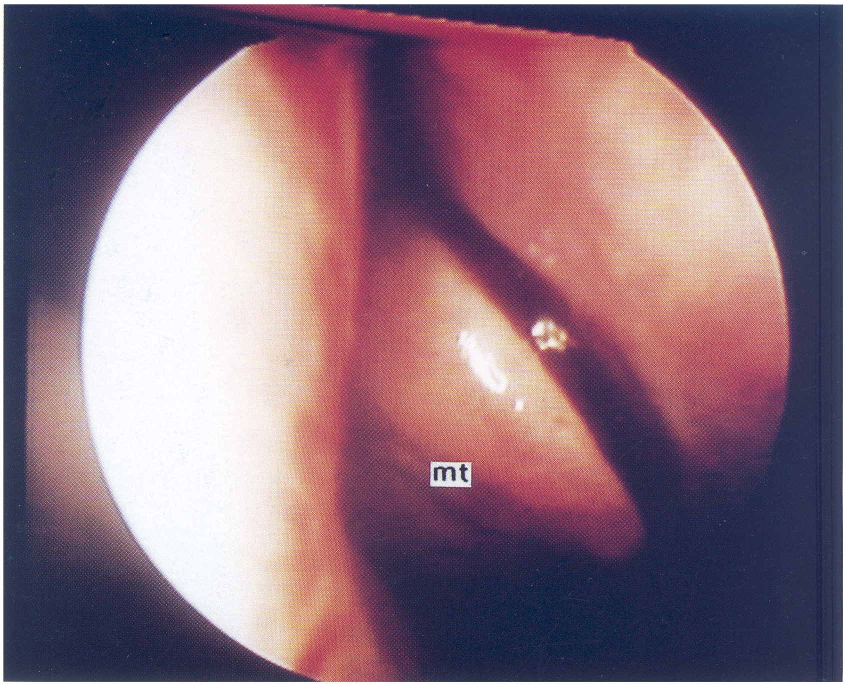

FIGURE 5 Concha bullosa.

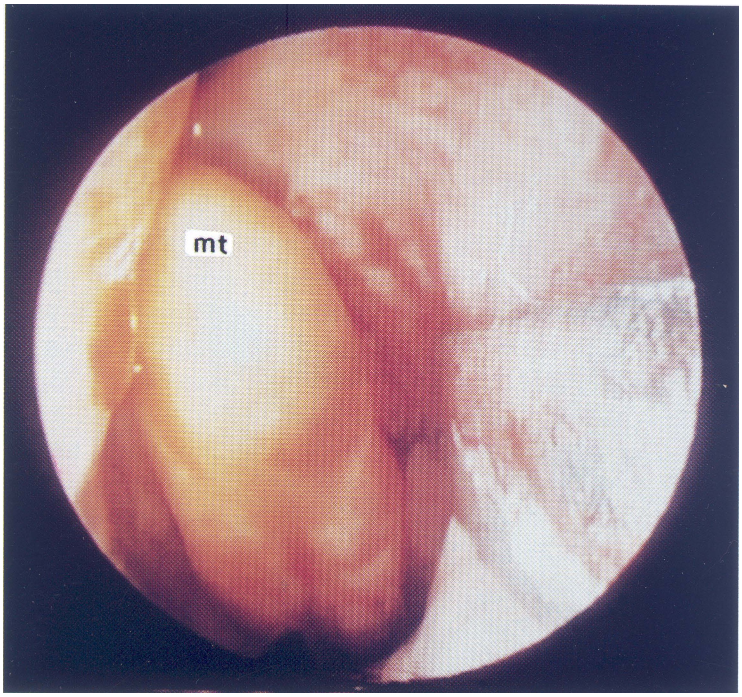

FIGURE 6 Sagittally clefted middle turbinate.

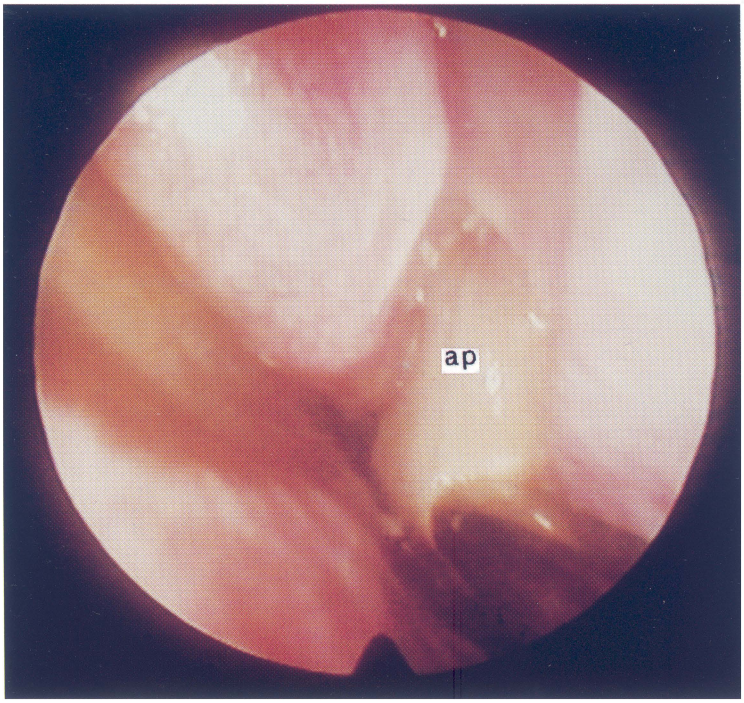

FIGURE 7 Left sided antrochoanal polyp.

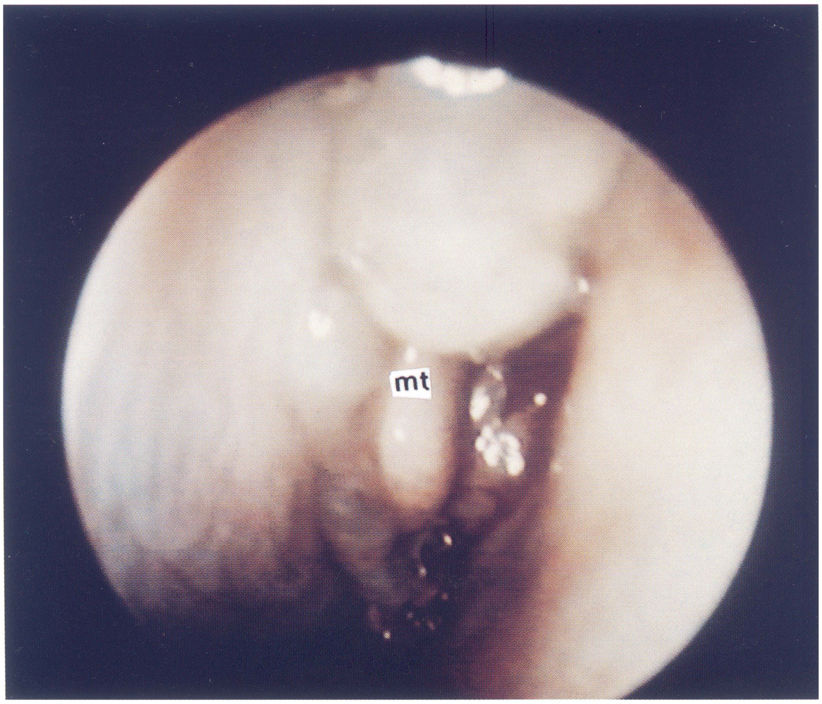

FIGURE 8 Massive nasal polyposis.

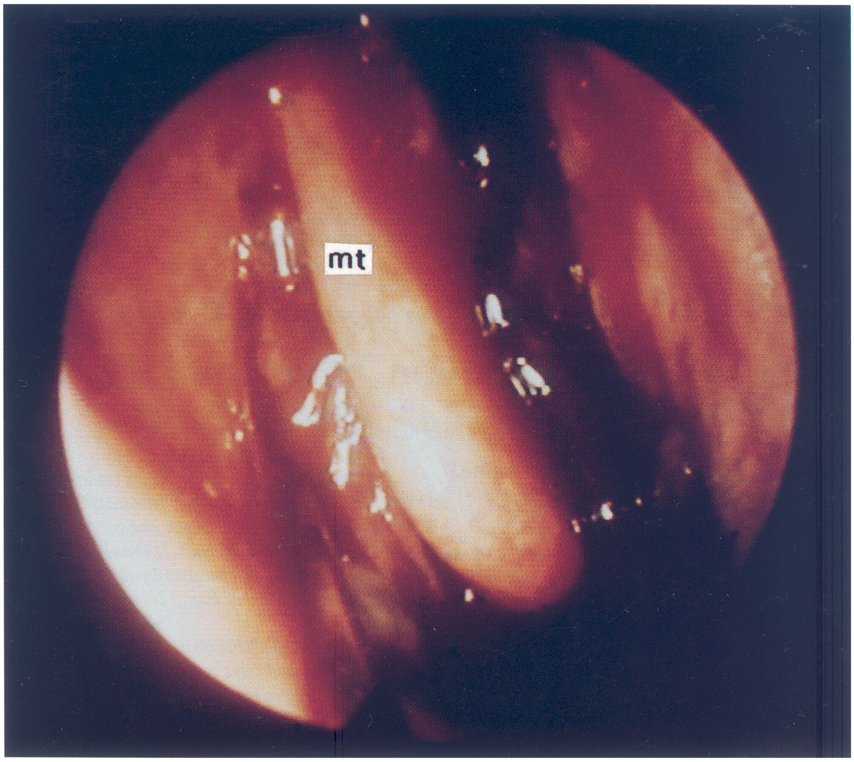

FIGURE 9 Normoplastic middle turbinate after removal of massive nasal polyposis.

### DISCUSSION

The middle turbinate (concha media) is an osseous shelf about 3.5–4 cm in length and is an important part of the ostiomeatal complex [5]. The permanent middle turbinate develops from the second ethmoturbinal and is an extension of the superior ethmoid bone covered with soft tissue and nasal mucosa [6]. From the anatomic point of view the middle turbinate has a conchal neck and a conchal head. Its anterior third is attached to the cribriform plate while its middle third is fixed to the lamina papyracea by the ground lamella. Its posterior third is fixed to the lateral wall and/or to the lamina papyracea [7]. Different anatomic variations were described. From "triangular or L-shaped" to second middle turbinate or sagittal groove. A sagittally clefted middle turbinate probably results from an interruption in its maturation process [8]. The most frequent anatomic variation encountered is a pneumatized middle turbinate or concha bullosa which occurs usually bilaterally. Pneumatization of the attachment of the middle turbinate is referred to as an interlaminary cell [9]. Another frequently found variation is a paradoxically bent middle turbinate [7]. However at the international conference on sinus disease in Princeton, anatomic variants do not require specific nomenclature apart from few exceptions e.g. concha bullosa [10]. Anatomic variations such as massive concha bullosa which contacts the lateral nasal wall, can interfere with mucociliary drainage of the ostiomeatal unit by inhibiting the cilial activity and so predispose to recurrent infections [11]. The middle turbinate which is an important landmark in sinus surgery has different functions including deflection of inspired air toward the olfactory epithelium [12]. It protects the structures of the lateral nasal wall and plays a role in warming and humidifying the inhaled air. Messerklinger distinguished three types of mucosal changes of the middle concha. The front end and free margin of the middle turbinate are particularly liable to hyperplasia. A diffuse hyperplasia of the conchal head can sometimes completely fill the space between the side wall of the nose and the nasal septum. The front end of a normoplastic middle concha is conspicuous as the "conchal head". With a more severe hyperplasia the conchal sinus and the meatus can be filled with polyps [13]. In most cases, antrochoanal polyps bulge out from the maxillary sinus usually through an accessory ostium, fill the floor of the nose and reach the choana [6,13].

### CONCLUSION

It seems that in most cases of chronic rhinosinusitis needing extensive sinus surgery, the middle turbinate can still be recognized and serve as a useful landmark. The most frequent mucosal change due to chronic inflammation seems to be polypous degeneration followed by hyperplastic mucosa. Still, in more than 30% the mucosa is surprisingly well preserved and normoplastic. Anatomical variations are not seen frequently 18%, the most common being paradoxically bent middle turbinate and concha bullosa.

### *References*

[1] Rice, D.H. and Schaefer, S.D. *Endoscopic Paranasal Sinus Surgery*. Raven Press, New York, 1988.
[2] Yanagisawa, E. and Weaver, E.M. Anatomical variations of the middle turbinate. *Ear, Nose & Throat Journal* 1996; **75**: 194–197.
[3] Ritter, F.N. and Arbor, A. The middle turbinate and its relationship to the ethmoidal labyrinth and the orbit. *Laryngoscope* 1982; **92**: 479–482.
[4] McCaffrey, T.V. *Rhinologic Diagnosis and Treatment*. Thieme, New York–Stuttgart, 1997.
[5] Ritter, F.N. and Fritsch, M.H. *Atlas of Paranasal Sinus Surgery*. Igaku-Shoin, Tokyo–New York, 1992.
[6] Stammberger, H.R. *Functional Endoscopic Sinus Surgery*. B.C. Decker, Philadelphia, 1991.
[7] Yanagisawa, E. Endoscopic view of the middle turbinate. *Ear, Nose & Throat Journal* 1993; **72**: 725–727.
[8] Rossiter, J.L. Sagittally-clefted middle turbinate: an endoscopic view. *Ear, Nose & Throat Journal* 1995; **74**: 452–453
[9] Anon, J.B., Rontal, M. and Zinreich, S.J. *Anatomy of the Paranasal Sinuses*. Thieme, New York, 1996.
[10] Stammberger, H.R. and Kennedy, D.W. Paranasal sinuses: anatomic terminology and nomenclature. *Annals of Otology, Rhinology & Laryngology* (*Suppl. 167*) 1995; **104**: 10.
[11] Bolger, M.W.E. and Kennedy, D.W. Nasal endoscopy in the outpatient clinic. *Otolaryngologic Clinics of North America* 1992; **25**: 791–802.
[12] Blaugrund, S.M. The nasal septum and concha bullosa. *Otolaryngologic Clinics of North America* 1989; **22**: 291–306.
[13] Messerklinger, W. *Endoscopy of the Nose*. Urban & Schwarzenberg, Baltimore–Munich, 1978.

